# Methylene Blue Modulates Transendothelial Migration of Peripheral Blood Cells

**DOI:** 10.1371/journal.pone.0082214

**Published:** 2013-12-10

**Authors:** Isabella Werner, Fengwei Guo, Nicolai V. Bogert, Ulrich A. Stock, Patrick Meybohm, Anton Moritz, Andres Beiras-Fernandez

**Affiliations:** 1 Department of Thoracic and Cardiovascular Surgery, Johann-Wolfgang-Goethe University Hospital, Frankfurt/Main, Germany; 2 Clinic of Anaesthesiology, Intensive Care Medicine and Pain Therapy, Johann-Wolfgang-Goethe University Hospital, Frankfurt/Main, Germany; Bristol Heart Institute, University of Bristol, United Kingdom

## Abstract

Vasoplegia is a severe complication after cardiac surgery. Within the last years the administration of nitric oxide synthase inhibitor methylene blue (MB) became a new therapeutic strategy. Our aim was to investigate the role of MB on transendothelial migration of circulating blood cells, the potential role of cyclic cGMP, eNOS and iNOS in this process, and the influence of MB on endothelial cell apoptosis. Human vascular endothelial cells (HuMEC-1) were treated for 30 minutes or 2 hours with different concentrations of MB. Inflammation was mimicked by LPS stimulation prior and after MB. Transmigration of PBMCs and T-Lymphocytes through the treated endothelial cells was investigated. The influence of MB upon the different subsets of PBMCs (Granulocytes, T- and B-Lymphocytes, and Monocytes) was assessed after transmigration by means of flow-cytometry. The effect of MB on cell apoptosis was evaluated using Annexin-V and Propidium Iodide stainings. Analyses of the expression of cyclic cGMP, eNOS and iNOS were performed by means of RT-PCR and Western Blot. Results were analyzed using unpaired Students T-test. Analysis of endothelial cell apoptosis by MB indicated a dose-dependent increase of apoptotic cells. We observed time- and dose-dependent effects of MB on transendothelial migration of PBMCs. The prophylactic administration of MB led to an increase of transendothelial migration of PBMCs but not Jurkat cells. Furthermore, HuMEC-1 secretion of cGMP correlated with iNOS expression after MB administration but not with eNOS expression. Expression of these molecules was reduced after MB administration at protein level. This study clearly reveals that endothelial response to MB is dose- and especially time-dependent. MB shows different effects on circulating blood cell-subtypes, and modifies the release patterns of eNOS, iNOS, and cGMP. The transendothelial migration is modulated after treatment with MB. Furthermore, MB provokes apoptosis of endothelial cells in a dose/time-dependent manner.

## Introduction

Vasoplegia or vasoplegic syndrome is a recognized and relatively frequent complication after cardiac surgery with cardiopulmonary bypass (CPB) with an incidence ranging between 8% and 25% [1], [2]. It is characterized by a multitude of signs and symptoms, including severe hypotension, decreased systemic vascular resistance, arteriolar reactivity as well as increased requirements for volume and vasopressive therapy, despite adequate cardiac output [3], [4]. It has been hypothesized that vasoplegia is caused by dysregulation of endothelial homeostasis and subsequent endothelial dysfunction and/or by direct and indirect effects of multiple inflammatory mediators [5]. Currently, conventional pharmacological therapy in the treatment of intraoperative or postoperative vasoplegia includes the administration of norepinephrine, phenylephrine and vasopressin [6]–[10] to maintain an adequate perfusion [11].

Several different mechanisms are believed to be causative to vasoplegia. The nitric oxide (NO)/cyclic guanosine 3′,5′ monophosphate (cGMP) pathway seems to play a prominent role [12]. It has been suggested that vasoplegia may be caused by a dysregulation of NO synthesis and vascular smooth muscle cell guanylate cyclase activation [4]. Two different types of NO synthase, a constitutive type and an inducible type, are known to produce nitric oxide. iNOS is mainly produced in vascular smooth-muscle cells [13] and cardiac myocytes [14]. The produced nitric oxide activates cGMP, which subsequently causes vasodilatation in smooth muscle cells [15] and possibly decreases contractility in myocytes [14]. *In vitro* studies have shown that NO functionally antagonizes the effects of the vasoconstrictors released during anaphylaxis [16] as well as that NO production might reduce some pathophysiological changes associated with anaphylaxis, except for vasodilatation [17].

An interesting therapeutic alternative to treat vasoplegic syndrome that has emerged within the last years is the administration of the guanylate cyclase and nitric oxide synthase (NOS) inhibitor methylene blue (MB). Methylene blue is a tricyclic phenothiazine drug which was synthesized in 1876 [5] and since then used in laboratories and clinics. Today, MB is recommended to be used to treat methemoglobinemia, vasoplegic syndrome, ifosfamide-induced encephalopathy and cyanide poisoning [18] by the US FDA. Nausea and vomiting, chest pain, dyspnea, and hypertension belong to the adverse events observed in humans after MB administration and are reported to be predominantly dose dependent [3], [19].

It is proposed that MB acts through competition with nitric oxide, by binding to the iron heme-moiety of soluble guanylate cyclase causing enzyme activation. The main effect of MB is thus related to an inhibition of the NO-mediated smooth muscle relaxation, causing a smooth muscle-related vasoconstriction. However, an early study on MB showed that endothelium-dependent relaxation of isolated blood vessels was noticeably more sensitive to inhibition by MB than relaxation induced by direct soluble guanylyl cyclase activators, suggesting an endothelium-dependent mechanism of action of MB [20].

Transendothelial migration and leukocyte trafficking are indispensable processes in inflammatory reactions. Vasoplegia, characterized by profound vasodilatation, is often associated to systemic inflammatory response (SIRS), including endothelial dysfunction [21]. We investigated the effect of MB upon the migration potential of circulating cells through an endothelial monolayer. Furthermore, the apoptosis of endothelial cells after different dosages and exposure times of MB was measured, to investigate the potential toxic effect of MB on endothelial cells.

## Materials and Methods

### Ethics Statement

This study was approved by the ethics committee on human research of the University of Frankfurt, Germany (GN: 189/13). All blood samples were obtained under informed consent and according to the declaration of Helsinki. All participants provided their written informed consent to participate in this study.

### Cell culture

Human microvascular endothelial cells-1 (HuMEC-1) [22] were kindly provided by Dr. V. Mirakaj (University Tübingen, Department of Anesthesiology and Intensive Care Medicine) and were cultured in MCDB-131 (Life Technologies, Darmstadt, Germany) supplemented with 10% fetal calf serum (Gibco, Karlsruhe, Germany), 1% glutamine (Gibco, Karlsruhe, Germany), 1% pen/strep solution (Sigma Chemical Co. St. Louis, USA), 10 ng/ml Epidermal Growth Factor (Sigma Chemical Co. St. Louis, USA) and 1 µg/ml hydrocortisone (Sigma Chemical Co. St. Louis, USA).

Jurkat cells [23] were cultured in RPMI-1640 medium (Sigma Chemical Co. St. Louis, USA) supplemented with 1% pen/strep solution and 10% fetal calf serum. Cells were cultured at 37°C and 5% CO_2_ atmosphere.

### Isolation of peripheral blood mononuclear cells (PBMCs)

Blood was drawn into EDTA tubes from healthy male and female volunteers (n = 5). Throughout the isolation process the samples were kept between 18–22°C. For density gradient centrifugation, the fresh blood samples were underlaid with 5 ml Polymorphprep® (Axis-Shield, Oslo, Norway) and centrifuged at 450 g for 35 minutes without break. The PBMC layer was aspirated and washed twice with PBS (Gibco, Karlsruhe, Germany). Finally PBMCs were resuspended in PBS, counted using a Neubauer chamber and used for further experiments.

### Treatment of HuMEC-1

HuMEC-1 were seeded in a density of 1×10^5^ cells/culture dish. After 24 h, HuMEC-1 were either treated with MB or LPS (Sigma Chemical Co. St. Louis, USA) alone or with MB followed by LPS and vice versa. The time intervals and concentration of the treatment procedure are summarized in Table S1. After treatment, supernatants were collected and total RNA was isolated from the cells and used for further analysis.

### Assessment of endothelial cell apoptosis

Endothelial cell apoptosis was determined by Annexin-V positive and propidium iodide (PI) negative staining. HuMEC-1 were plated in 6-well plates and incubated at 37°C in a 5% CO_2_ atmosphere. After 24–28 hours, they were treated with 10 µM, 30 µM or 60 µM MB. After 30 min or 120 min of treatment, cells were gently detached with Alfazyme (PAA, Pasching, Austria) and rapidly stained with an APC-labeled Annexin-V Detection Kit APC (eBioscience, San Diego, USA) according to the manufacturer's instructions. Sample tubes were acquired and analyzed on a FACSCantoII flow cytometer with FACSDiva 6.1.2 software (BD, Heidelberg, Germany).

### Transmigration assays

Transmigration assays were assessed with the use of 3-µm pore size Fluoroblok inserts (BD, Heidelberg, Germany) in triplicate. BrdU-labelled non-proliferating HuMEC-1 were placed in fibronectin-coated inserts and allowed to form an endothelial monolayer for approximately 24 h. HuMEC-1 were either treated with MB or LPS alone or with MB followed by LPS and vice versa. The time intervals and concentration of the treatment procedure are summarized in Table S1. Jurkat cells or PBMCs stained with calcein-AM (ABD-Bioquest, Biomol, Hamburg, Germany) were suspended in assay medium (RPMI−1640+0.5% BSA) and applied on top of the inserts and were allowed to transmigrate. The use of Jurkat cells is widely accepted to study human t-lymphocyte reactions. As the relative proportion of T-lymphocytes in PBMCs can range from 61–85% [24] in healthy adults, they represent the predominant cell population within PBMCs. Using this cell line gave us the opportunity to investigate the potential effect of MB on not activated t-lymphocytes as well as excluding circulating cell interaction of other leukocyte sub-types. To ensure adequate assay performance, a positive control, where 50 ng/ml CXCL12 (R&D Systems, Wiesbaden, Germany) was added to the lower chamber compartment was performed (Data not shown). After 2 hours at 37°C, the fluorescence signal of cells migrated to the bottom surface of the filter was measured with a microplate reader and expressed as percent of control (transmigration index, tmx). To inhibit transendothelial migration potentially regulated by eNOS and iNOS, 100 µM L-NMMA (biomol, Hamburg, Germany), a known inhibitor of both iNOS and eNOS, was added to the top of the transwell-filter during the 2 h lasting transmigration experiment. Transmigration index was calculated as percent of controls.

### Differentiation of transmigrated PBMC subtypes

Freshly isolated PBMCs were stained previously to the transmigration experiments with fluorescence labeled antibodies directed against CD3 (Brilliant Violet conjugated), CD19 (PECy7 conjugated), CD14 (APC conjugated), CD16 (PE, conjugated, all from BD Bioscience, Heidelberg, Germany) and CD177 (FITC conjugated, Biolegend, San Diego, USA). After transendothelial migration, the migrated cells below the transwell-filter were collected, washed and suspended in FACS staining buffer (BD, Bioscience, Heidelberg, Germany). PBMC subtypes were analyzed with a FACSCantoII flow cytometer with FACSDiva 6.1.2 software (BD, Heidelberg, Germany) and FlowJo software. The PBMC subtype gating strategy is presented in Figure S1.

### Enzyme-Linked Immunoadsorbent Assay (ELISA)

cGMP levels in the supernatants of the treated HuMEC-1 were determined using total cyclic GMP Enzyme Immunoassay Kit from Biotrend (Köln, Germany) in accordance with the manufacturer's protocol. To increase the kits' sensitivity, the acetylated format was used.

### Total RNA isolation, cDNA synthesis and RT-PCR

Total RNA was isolated from HuMEC-1 with ZR RNA MiniPrep™ Kit (Zymo Research, Irvine, U.S.A.) according to the manufacture's protocol. Total RNA concentration was measured with nanodrop (Nano Vue, General Electric, Connecticut, USA). Appropriate quantity of cDNA was reverse transcribed with High Capacity cDNA Reverse Transcription Kit with RNase Inhibitor (Life Technologies, Applied Biosystems, Darmstadt, Germany) according to the manufacture's protocol. RT-PCR was performed using specific oligonucleotide primers for iNOS and eNOS (both SABioscience, Hilden, Germany) using MX3005P PCR cycler (Stratagene, Santa Clara, USA). GAPDH served as housekeeping gene for the comparisons of gene expression data of iNOS and eNOS (SABioscience, Hilden, Germany).

### Western blot analysis

To analyze eNOS, iNOS and their phosphorylated form proteins in HuMEC-1, cell lysates were applied to a 7% polyacrylamide gel and electrophoresed (90 min, 60 V followed by 100 V). The proteins were then transferred to nitrocellulose membranes (1 h, 100 V). The membranes were blocked with non-fat dry milk for 1 h, and then incubated overnight with polyclonal antibodies directed against eNOS (Thermo Scientific, Rockford, USA), iNOS (Thermo Scientific, Rockford, USA), phospho-eNOS pSer1177 (Thermo Scientific, Rockford, USA) and iNOS Tyr-1055 (ECM Bioscinece, Versailles, USA). HRP-conjugated goat-anti-rabbit IgG and HRP-conjugated goat-anti-mouse IgG (both Millipore, Temecula, CA, USA) served as the secondary antibody for eNOS, iNOS, phospho-eNOS pSer1177, iNOS Tyr-1055 and β-actin, respectively. The membranes were briefly incubated with ECL detection reagent (ECL™, Amersham/GE Healthcare, München, Germany) to visualize the proteins and then analyzed by the Fusion FX7 system (Peqlab, Erlangen, Germany). β-actin (Sigma, Taufenkirchen, Germany) served as the internal control. The visualized protein bands were analyzed using ImageJ software. The relative amount of protein (rap) was calculated in relation to β-actin expression.

### Statistical analysis

Data are presented as mean ± SEM. Statistical analysis was performed with Prism 6 software (Graph Pad) using unpaired Students T-test. Differences with p<0.05 were considered statistically significant.

## Results

### Dose dependent increase of endothelial cell apoptosis

**Figure 1:**
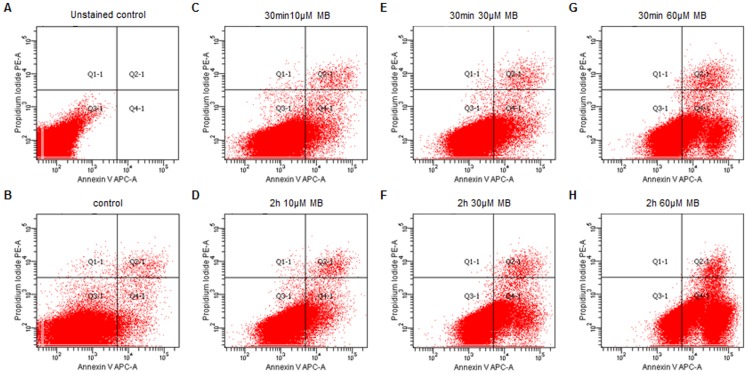
Endothelial cells apoptosis after MB treatment. The number of APC-positive/PI-negative HuMEC-1 after MB increased with higher MB dosages. (A) Unstained and untreated controls served as initial point for the gating strategy. (B) Untreated control. (C–D) HuMEC-1 treated with 10 µM MB for 30 min and 120 min. (E–F) HuMEC-1 treated with 30 µM MB for 30 min and 120 min. (G-H) HuMEC-1 treated with 60 µM MB for 30 min and 120 min. (each experiment n = 3)

Endothelial cell apoptosis was determined by Annexin-V positive and PI negative staining. The number of APC-positive/PI-negative HuMEC-1 increased with higher MB concentration, indicating a dose dependent apoptosis of endothelial cells. Treatment with 60 µM caused a significant increase of apoptosis compared to non-treated HuMEC-1 and low MB-dose treated HuMEC-1 (10 µM 30 min and 120 min) (Table 1, Figure 1).

**Table 1:** Dose dependent endothelial cell apoptosis.

Treatment	Concentration	Time [min]	APC positive/PI negative cells [%]
Non-treated control			3.15±0.64*
**MB**	10 µM	30	5.48±1.91*
		120	6.42±2.18*
	30 µM	30	9.60±2.99
		120	19.10±5.00
	60 µM	30	23.80±4.80
		120	52.05±9.85 *

The number of apoptotic human microvascular endothelial cells significantly increases in a dose dependent manner (120 min 60 µM vs. control, 30 min 10 µM and 120 min 10 µM) but remains stable regarding the incubation time (* p<0.05, data represent mean±SEM).

### Time and dose dependent effect of MB on transendothelial migration of blood circulating cells

#### Transendothelial migration of PBMCs

**Figure 2:**
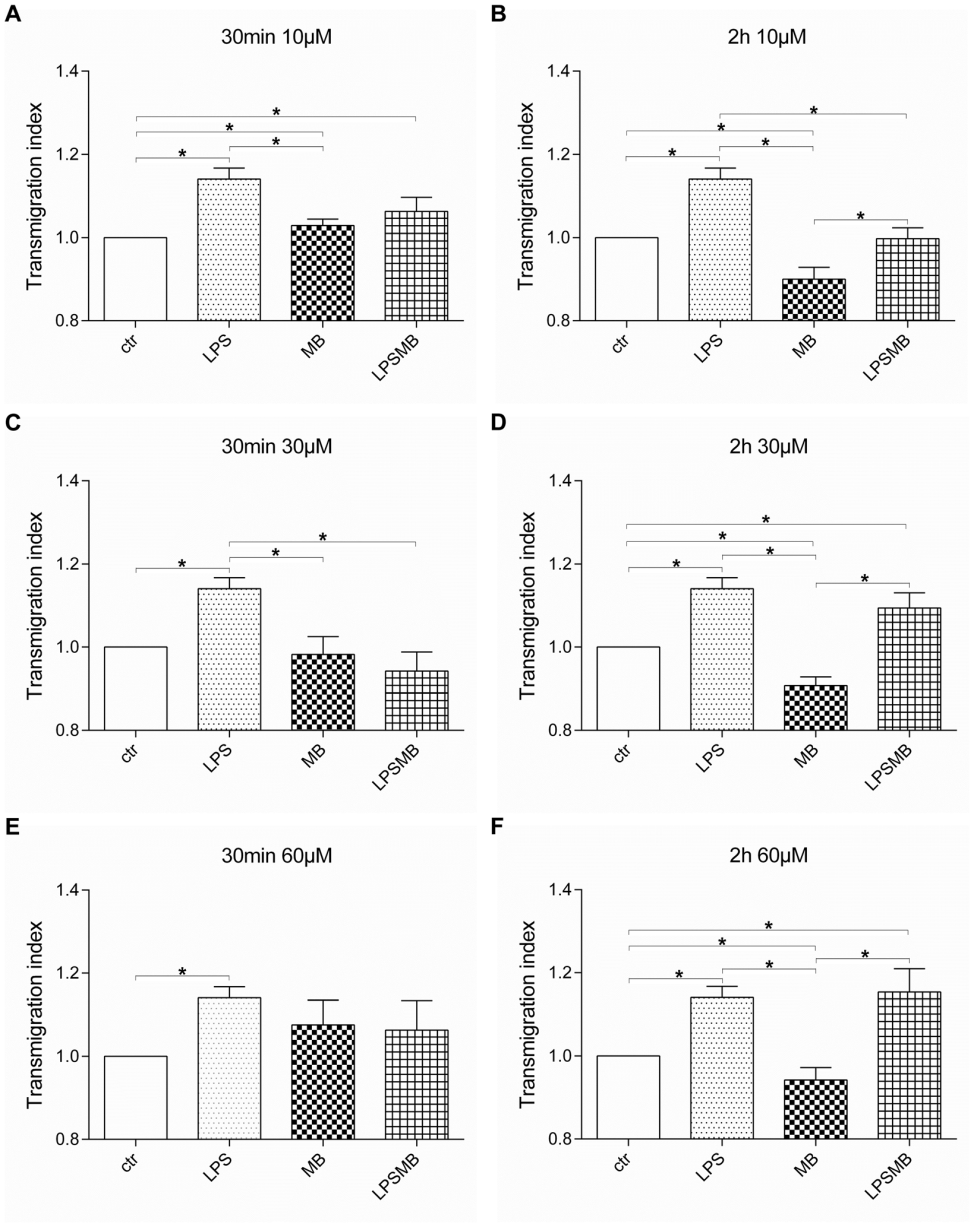
Influence of MB on transendothelial migration of PBMCs. Transmigration of PBMCs through an endothelial layer after treatment with (A) 10 µM MB for 30 min. (B) 10 µM MB for 2 h. (C) 30 µM MB for 30 min. (D) 30 µM MB for 2 h. (E) 60 µM MB for 30 min. (F) 60 µM MB for 2 h (each experiment n = 5–8) (*p<0.05).

After different treatment procedures (Table S1), PBMCs were allowed to transmigrate through a HuMEC-1 monolayer for up to 2 h. In Figure 2 the modulation of transmigration of PBMCs is summarized. MB caused a time but not dose dependent decrease of transmigrated PBMCs. The treatment of HuMEC-1 for 2 h, irrespectively of the concentration, led to a significant down-regulation of transmigration compared to control. Short time administration of MB did not cause a change in transendothelial migration at high concentration. A significant up-regulation at 30 min 10 µM MB was observed which was reversed in long term. LPS caused a significant up-regulation of transendothelial migration compared to controls in all experimental groups. MB administration after LPS significantly decreased the transmigrated PBMCs at 30 µM MB for 30 min and at 10 µM MB for 2 h. In the other experimental groups, transendothelial migration remained at same high levels as LPS stimulation of HuMEC-1 alone.

#### Transendothelial migration of Jurkat cells

**Figure 3:**
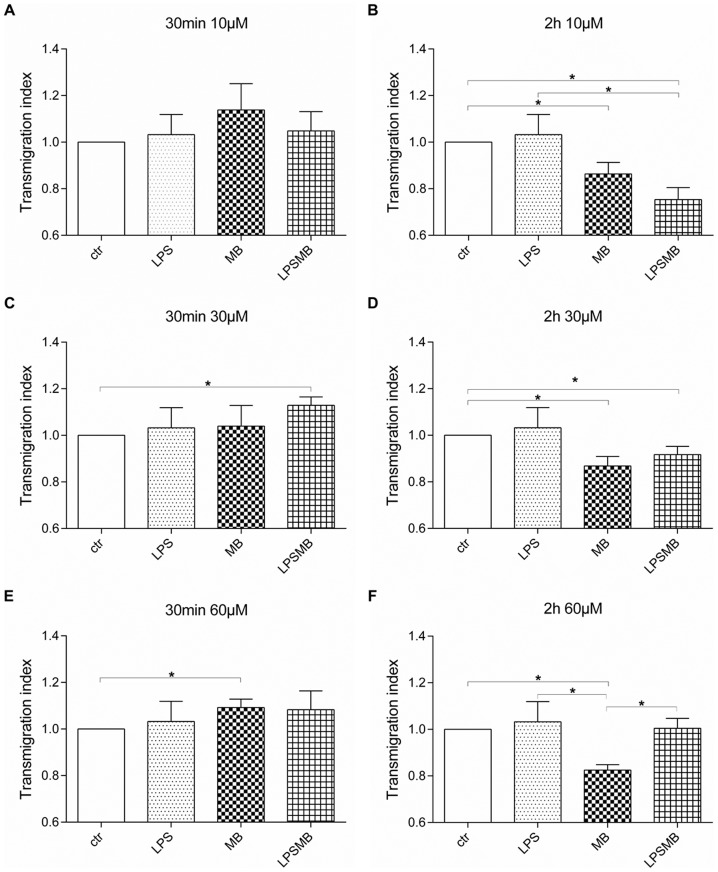
Influence of MB on transendothelial migration of Jurkat cells. Transmigration of Jurkat cells through an endothelial layer after treatment with (A) 10 µM MB for 30 min. (B) 10 µM MB for 2 h. (C) 30 µM MB for 30 min. (D) 30 µM MB for 2 h. (E) 60 µM MB for 30 min. (F) 60 µM MB for 2 h (each experiment n = 3–6) (*p<0.05).

In Figure 3 the modulation of transmigration index of Jurkat cells is summarized. Jurkat cells showed no change in transmigration behavior when HuMEC-1 were treated for 30 min with low concentrations of MB compared to controls. Compared to control, 60 µM MB for 30 min caused a significant increase of transmigration. However, when administration time with MB was prolonged for 2 h, transmigration rate of Jurkat cells was significantly diminished compared to control group. Incubation of HuMEC-1 with LPS did not affect transmigration rate of Jurkat cells. LPS stimulation followed by 10 µM MB for 2 h provoked a noteworthy decline in transmigrated cells compared to LPS triggered group and control. This significant down-regulatory effect is flattened when the MB dose increased.

### MB affects transmigration behavior of PBMC subtypes

**Figure 4:**
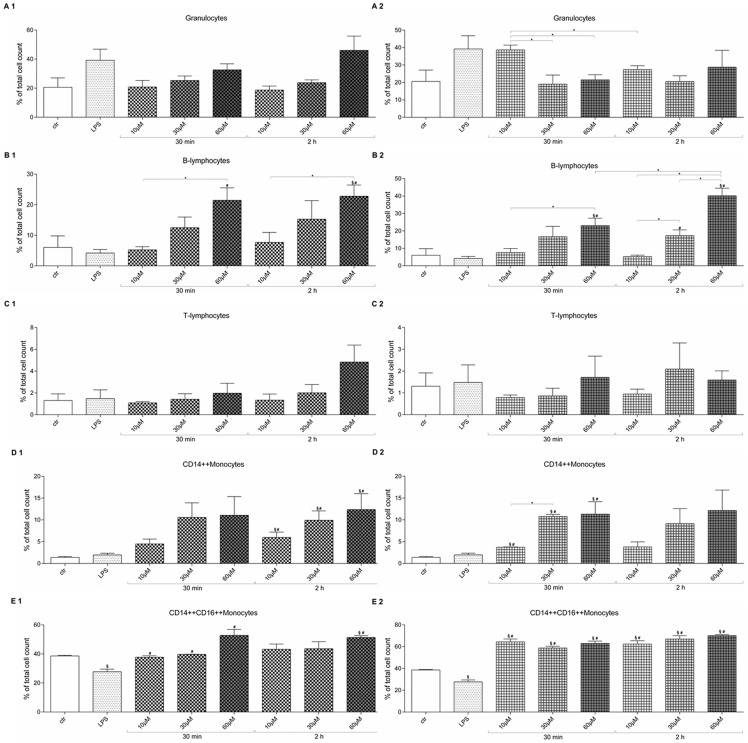
Change of migrated PBMC subtypes after MB and LPSMB treatment. (A1–E1) MB treatment of HuMEC-1 previous to transmigration assay. (A2–E2) LPSMB treatment of HuMEC-1 previous to transmigration assay. (A1, A2) Migrated granulocytes, (B1, B2) Migrated B-lymphocytes. (C1,C2) Migrated T-lymphocytes. (D1, D2) Migrated CD14++ monocytes. (E1,E2) Migrated CD14++CD16++ monocytes. (each experiment n = 3) (§ p<0.05 vs. control; # p<0.05 vs. LPS, *p<0.05).

The FACS analysis of migrated PBMC subtypes showed that the amount of transmigrated granulocytes does not change after MB treatment of endothelial cells (Figure 4 A1). In case of induced inflammation by LPS followed by MB treatment of endothelial cells, MB is able to significantly reduce the number of migrated granulocytes at higher MB doses for short time compared to low MB dose (Figure 4 A2). Significantly higher numbers of B-lymphocytes migrate after treatment of endothelial cells with high dosage MB compared to control, LPS and low MB dosages (Figure 4 B1). The same effect was observed when endothelial cells were treated with LPS followed by MB (Figure 4 B2). The amount of transmigrated T-lymphocytes did not change in any of the experimental groups (Figure 4 C1–2). Significantly more CD14++ monocytes transmigrated when the endothelial layer was treated with MB for 2 h compared to control and LPS group (Figure 4 D1). A similar trend can be seen at the 30 min MB groups without any significance. Endothelial cell treatment with LPS followed by MB also caused a dose dependent increase of CD14++ monocyte transmigration. Short term LPSMB treatment increased the number of migrated CD14++ monocytes significantly compared to control and LPS group. The 2 h LPSMB treatment only evoked a dose dependent elevation by trend without significance (Figure 4 D2). LPS treatment of the endothelium caused a significant reduction of CD14++CD16++ monocytes compared to control. The high dose MB treatment lead to a significant elevation of transmigrated CD14++CD16++ monocytes compared to control and LPS (Figure 4 E1). LPS treatment followed by MB exposure caused a significant up-regulation of transmigrated CD14++CD16++ monocytes in all groups compared to control and LPS group (Figure 4 E2).

### Prophylactic administration of MB leads to an increase of transendothelial migration of PBMCs but not Jurkat cells

**Figure 5:**
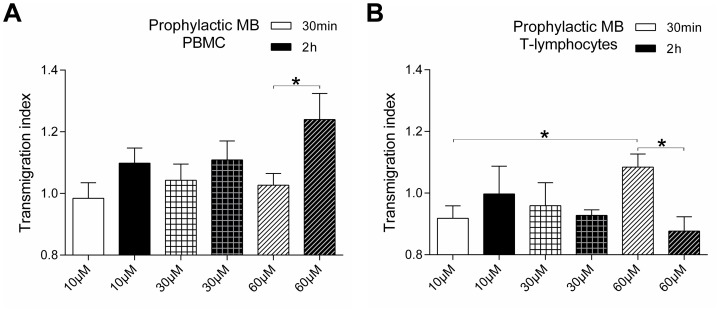
Prophylactic MB treatment previous to inflammation modulates transendothelial migration. (A) Transmigration of PBMCs through HuMEC-1 after prophylactic treatment with MB followed by LPS (n = 9). (B) Transmigration of Jurkat cells through HuMEC-1 after prophylactic treatment with MB followed by LPS (each experiment n = 4–8) (*p<0.05).

In case of MB administration followed by LPS a time dependent increase of transmigration activity of PBMCs can be observed (Figure 5 A). Only the dosage of 10 µM and 30 µM MB for 2 h show a higher transmigration trend of PBMCs compared to the 30 min administration. However, 60 µM MB cause a significant up-regulation in transmigrated cells. Comparing these effects to transmigration of Jurkat cells, the opposite effect can be observed (Figure 5 B). Short time incubation of HuMEC-1 with MB for 30 min followed by LPS leads to a dose dependent increase. In addition, long term incubation of 2 h with 60 µM MB caused a significant drop compared to the 30 min 60 µM MB group.

### Inhibitory effect of L-NMMA on transendothelial migration of PBMCs

**Figure 6:**
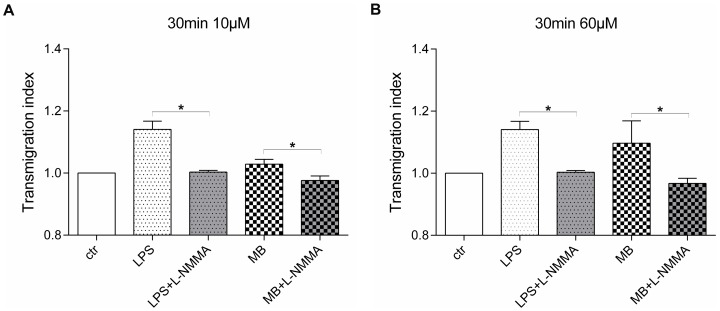
L-NMMA inhibits transendothelial migration after MB treatment. (A) Treatment of HuMEC-1 with either LPS or 10 µM MB for 30 min previous to transendothelial migration. L-NMMA was added during transmigration to LPS and MB group. (B) Treatment of HuMEC-1 with either LPS or 60 µM MB for 30 min previous to transendothelial migration. L-NMMA was added during transmigration to LPS and MB group. (each experiment n = 3–6) (*p<0.05)

The application of the specific NOS inhibitor L-NMMA shows a significant reduction of transendothelial migration after MB treatment (Figure 6). PBMCs enriched with L-NMMA significantly reduced their transmigration rate through HuMEC-1 treated with LPS previous to transendothelial migration experiment. The same significant reduction in transendothelial migration due to addition of L-NMMA can be seen in the 30 min 10 µM MB group as well as in the 30 min 60 µM MB group.

### HuMEC-1 secretion of cGMP correlates with iNOS expression after MB administration but not after LPSMB treatment

**Figure 7:**
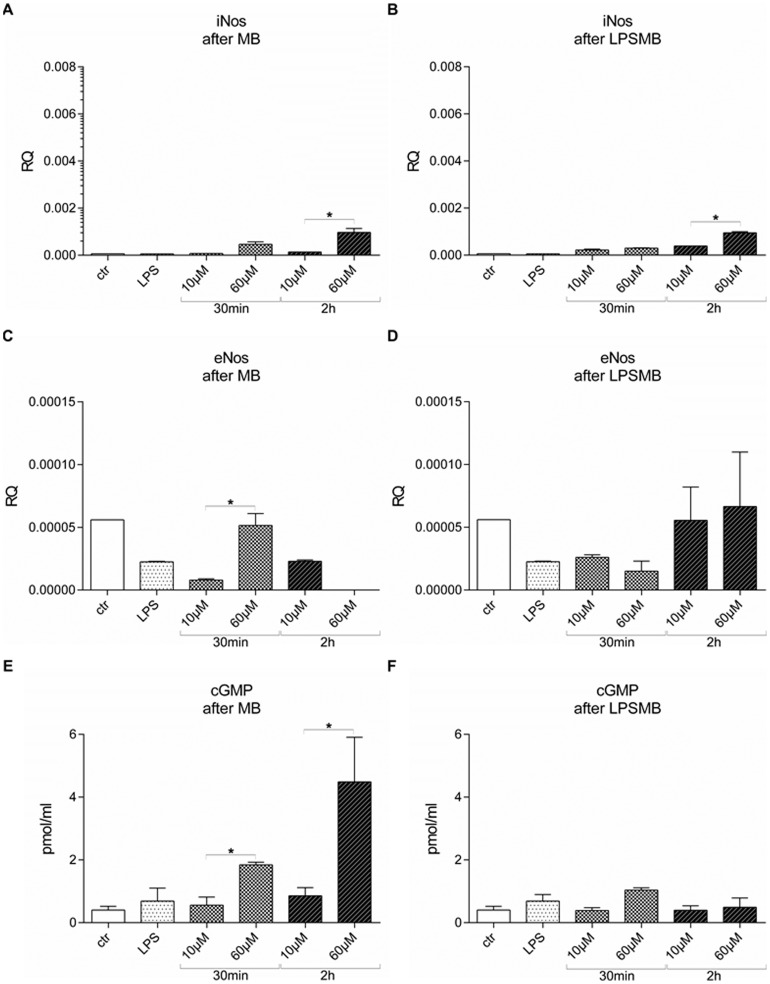
Modulation of eNOS and iNOS expression and secretion of cGMP of HuMEC-1 after MB and LPSMB administration. (A) iNOS expression is dose dependent elevated by MB administration compared to control. (B) Stimulation of HuMEC-1 with LPS followed by MB treatment increases iNOS expression after 2 h MB. (C) MB treatment of HuMEC-1 reduces eNOS expression. (D) LPS stimulation prior to MB administration reduces eNOS expression. (E) cGMP content in HuMEC-1 supernatants after different MB administrations. (F) Blocked cGMP released from the endothelial cells after LPSMB treatment. (each experiment n = 4) (*p<0.05).

Gene expression of eNOS and iNOS was analyzed using RT-PCR. Treatment of HuMEC-1 with MB caused a substantial drop of eNOS in the majority of the experimental groups (Figure 7 C). Dose dependent increase of eNOS expression is evoked in the 30 min incubation groups. In contrast, long term administration of MB for 2 h initiates a significant dose dependent decline of eNOS (Figure 7 C). However, iNOS expression was elevated by MB administration compared to control (Figure 7 A). Short incubation time of 30 min did not lead to a dose dependent modulation in iNOS gene expression. This fact changed when incubation time was prolonged to 2 h. Thus, a dose dependent increase of iNOS expression from 10 µM MB to 60 µM MB can be observed. The analysis of HuMEC-1 supernatants after different MB treatment protocols exhibits a dose dependent production of cGMP (Figure 7 E). An incubation time of 30 min and 2 h with different MB concentration triggered a dose dependent cGMP release from HuMEC-1. This observation correlated with iNOS expression when cells were treated with MB alone.

Stimulation of HuMEC-1 with LPS followed by MB treatment led to increased iNOS expression after 2 h MB (Figure 7 B). These results are similar to the observed changes when cells were treated with MB alone. The LPS stimulation prior to MB administration eliminated the dose and time responds of eNOS triggered by MB treatment alone (Figure 7 D). This treatment procedure additionally blocked the cGMP released from the endothelial cells after long term MB treatment (Figure 7 F).

### MB treatment of endothelial cells reduces iNOS and eNOS protein expression

**Figure 8:**
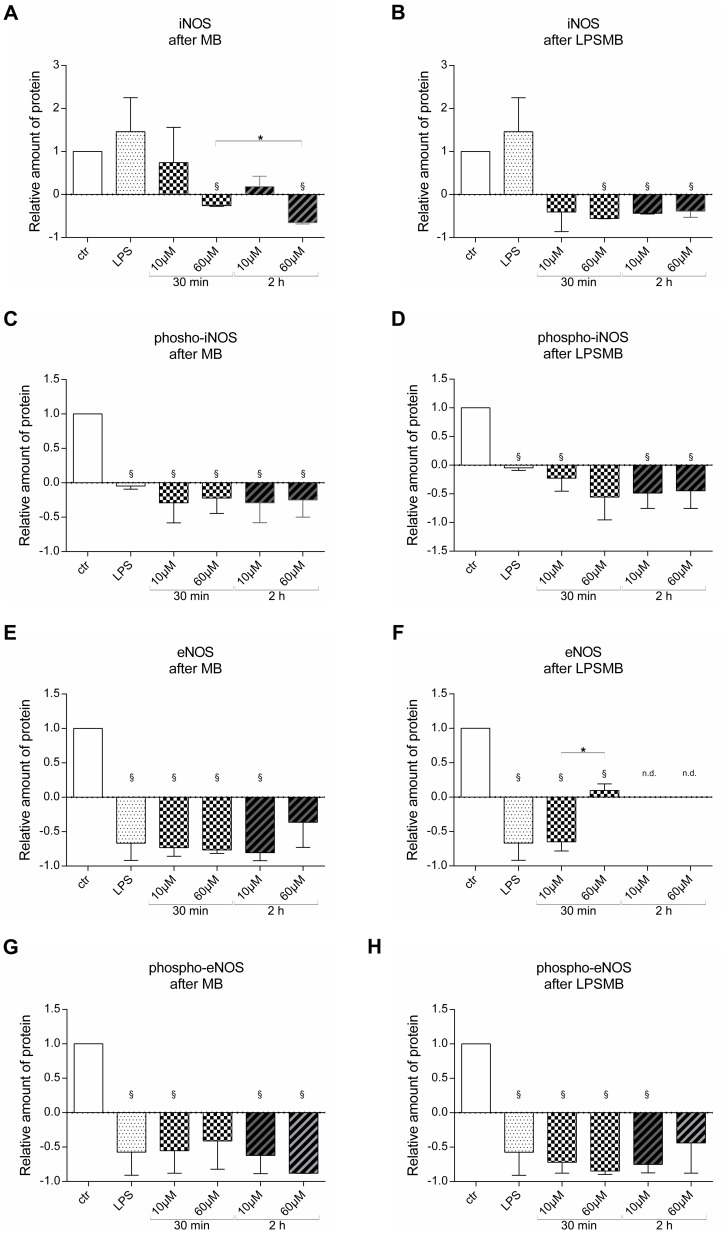
Protein expression levels of eNOS vs. phosphor-eNOS and iNOS vs. phosphor-iNOS. (A) Relative amount of iNOS protein expression after MB treatment. (B) Relative amount of iNOS protein expression after LPSMB treatment. (C) Relative amount of phosphorylated iNOS protein expression after MB treatment. (D) Relative amount of phosphorylated iNOS protein expression after LPSMB treatment. (E) Relative amount of eNOS protein expression after MB treatment. (F) Relative amount of eNOS protein expression after LPSMB treatment. (G) Relative amount of phosphorylated eNOS protein expression after MB treatment. (H) Relative amount of phosphorylated eNOS protein expression after LPSMB treatment. (each experiment n = 2-3) ((§ p<0.05 vs. control; *p<0.05).

Protein expression of iNOS, eNOS and their phosphorylated forms were analyzed using western blot analysis. Western blot experiments confirmed that iNOS expression of HuMEC-1 significantly drops after high dose MB treatment compared to control. Additionally, a time dependent reduction of iNOS protein was observed after high dose MB treatment (Figure 8 A). Induction of inflammation by LPS followed by MB treatment of HuMEC-1 caused a significant fall in iNOS protein expression compared to control at high dose and long term MB treatment (Figure 8 B). The phosphorylated form of iNOS significantly dropped in all experimental groups compared to control after MB treatment of HuMEC-1 (Figure 8 C). Similar low expression levels of phosphorylated iNOS are observed after LPSMB treatment of the endothelial cells (Figure 8 D). eNOS protein levels of HuMEC-1 significantly drop after MB treatment in the 30 min group and the low dose 2 h group compared to control (Figure 8 E). In case of 30 min LPSMB treatment of HuMEC-1, a significant dose dependent increase in eNOS expression can be observed. Any eNOS protein was detected after long term treatment for 2 h with LPSMB (Figure 8 F). The phosphorylated form of eNOS was only found in very low levels after MB as well as after LPSMB treatment of HuMEC-1. After MB treatment significant reduction was observed in the LPS, 30 min 10 µM MB, 2 h 10 µM MB and 2 h 60 µM MB group (Figure 8 G). After LPSMB treatment significant reduction was observed in the LPS, 30 min 10 µM MB, 30 min 60 µM MB and 2 h 30 min MB group (Figure 8 H). The normalization of phosphorylated eNOS to non-phosphorylated eNOS after MB treatment shows a dose but not time dependent elevation. On the other hand, a dose but not time dependent reduction was observed when normalizing phosphorylated iNOS to non-phosphorylated iNOS after MB treatment.

## Discussion

Catecholamine refractory hypotension is a severe complication after cardiac surgery with cardiopulmonary bypass, with an incidence of 10% [25]. Levin et al. analyzed 56 patients with vasoplegic syndrome and showed minor morbidity and mortality rates after treatment with MB [2]. Methylene blue is a thiazine dye [26] and can influence a diversity of pharmacologic effects, such as inhibition of monoamine oxidase [27], reduction of methemoglobin [28], inhibition of both guanylate cyclase [20] and nitric oxide synthase [29]. MB has been used in clinical setting to help the diagnosis of fistulae, and also in endoscopic diagnostic to visualize tissue structures [30], [31], because MB crosses cell membranes pretty fast and is able to stain living cells for a short term. Recent pharmacokinetic studies have shown that MB can accumulate and cause dose dependent toxic effects on the central nervous system [32]. MB has also shown to be preferential cytotoxic to tumor cells compared to PBMCs obtained from healthy volunteers [33]. Chang et al. have stated that MB cause a dose dependent damage to corneal endothelial cells, providing a rationale for the study of endothelial cell apoptosis after MB, which may be responsible for increased leukocyte trafficking and clinical inflammation [34]. Our data on endothelial cell apoptosis confirm these previous studies. MB shows a dose dependent cytotoxic effect on microvascular endothelial cells. Furthermore, based on our results, the exposure time of MB on endothelial cells seems to play only a subordinate role in the development of apoptosis. Apoptosis is intertwined with pro-inflammatory responses [35]. The increase of apoptotic endothelial cells may cause an elevation of pro-inflammatory mediators and trigger more circulating cells to transmigrate into tissue. Apoptotic endothelial cells could also lead to leakage formation in the inner vessel wall and facilitate the migration of PBMCs.

The role of MB in transendothelial migration of peripheral blood cells has not been clarified yet. Mayer et al. provided evidence, that stimulation of purified soluble GC led to a reduction of cGMP formation of 50% at approximately 60 µM MB. Hence, MB seems to act primarily via inhibition of NO synthase, with enzyme-bound heme being a possible target in its inhibitory action [20]. Nitric oxide is a signaling molecule involved in human immune response regulation which also includes the processes of adhesion and migration of leukocytes [36], [37]. Data from Carreau et al. provided evidence that NO in an optimal concentration contribute to angiogenesis, which includes the adhesion and transmigration processes [38], but lower or higher concentration can cause a reduction or even inhibition of this process [39]. Thus, the inhibition of NO via MB may lead to increased adhesion, followed by transmigration. We were able to show that the inhibition of NOS by its specific inhibitor L-NMMA, significantly reduced transendothelial migration of PBMCs through MB treated endothelial cells. These effects are of special interest after LPS administration, after which MB in low or medium dosage reduced the transmigration. This fact would add significance to the data from Carreau et al., confirming that the NO system is responsible for the modulation of cellular transmigration observed after MB treatment.

It has been shown that the inhibition of NO may affect the adhesion molecules responsible for transmigration of T-Lymphocytes, such as ICAM-1 [40], [41] and VCAM-1 [42], [43]. However, the expected increase or at least constant transmigration of cells when blocking NO with MB failed to appear. Thus, the inhibition of NO via MB may not affect these adhesion molecules. In case of inflammation, Jurkat cells reduce their transmigration eager compared to control. PBMCs, on the other hand, clearly up-regulated their transmigration rate in an inflammatory environment compared to MB administration alone and controls. Our results are probably related to a shift in the cellular populations implicated in the transmigration processes, indicating that monocytes adopt a more crucial role when inflammation occurs. The subtype analyses of PBMCs showed an increase in transmigration of CD14+/CD16+ monocytes after treatment with MB, partly confirming this hypothesis. Furthermore, the FACS analysis showed that the CD14+ monocyte and granulocyte response after MB treatment is dose-dependent, and T-lymphocytes showed a heterogeneous response, suggesting a predominant role in migration of monocytes and granulocytes after MB administration. The potential alteration of the PBMCs circulating pool after MB administration should be investigated in a clinical setting, to assess the effects observed in patients. As the potential contribution of the differential migration of PBMC subpopulations in inflammation is not fully clarified today [44], this mechanism should be further analyzed.

Several studies have investigated the prophylactic MB administration in a clinical setting to reduce the incidence of vasoplegia, showing a lower rate of transfusions and vasopressor requirements [3], [45]. The prophylactic administration of MB in our experimental model showed that the concentration of MB seems to play a predominant role. Furthermore, the time point when MB should be injected needs to be further investigated in a clinical setting as it seems that circulating blood cells respond to prophylactic MB in a time and dose dependent manner.

Previous observations postulated that NO inhibits monocyte function via a cGMP-mediated mechanism *in vitro* [46]. It has been shown that NO synthase inhibitors significantly attenuate chemokine triggered monocyte chemotaxis [47]. This inhibitory effect was reversed by addition of membrane permeable cGMP analog, indicating a prominent role of the NO/cGMP pathway in the regulation of human monocyte locomotion during inflammation. Our results confirm the modulation of monocyte transmigration after MB administration. It has also been postulated that MB, as an inhibitor of guanylate cyclase (GC), may decrease the level of its product cGMP in rat thoracic aorta tissue [48]. The depressed systemic vascular resistance characteristic for irreversible hemorrhagic and septic shock, is thought to be caused by excessive NO production [49], [50]. Vasodilatation by NO is cGMP-dependent and thus the therapeutic option of inhibiting GC via MB was proposed [51]. Our Western Blot data show that MB treatment alone in low doses does not block iNOS expression thus allowing a high cGMP release by the endothelial cells. As eNOS is less expressed after MB administration, we postulate that iNOS but not eNOS modifies cGMP release of endothelial cells after MB. In case of inflammation prior to MB administration the modulating role of iNOS and eNOS seems to be changed. It seems that MB blocks eNOS but not iNOS in inflammatory environment and thus leads to reduced cGMP production and thus to the aspired therapeutic effect of MB. These hypotheses are partly confirmed by our protein data, in which LPS is able to induce a higher expression of iNOS, but not eNOS. The discordance of the protein expression of iNOS and eNOS with the RT-PCR results may be explained by the increased rate of apoptosis of the endothelial cells.

The use of MB in the clinical setting is poorly understood. Our data, especially regarding the prophylactic administration of MB, highlight the dose and time dependent modulation of circulating blood cells after MB treatment. Particularly with regard to patients with an inflammatory profile we have found that the interplay of inflammation and MB administration may severely affect the migration behavior of PBMCs and thus may further enhance inflammatory reactions. Furthermore, our results confirm that the time point of MB administration is crucial, both as prophylaxis and as treatment in vasoplegic syndrome.

## Conclusion

This study gives new insights into the effect of MB on transendothelial migration, and apoptosis of endothelial cells in an *in-vitro* model. Our data clearly show that the endothelial response to MB is dose- and especially exposure time-dependent. MB shows different effects on circulating blood cell subtypes, and influences the release patterns of eNOS, iNOS, and cGMP. In an inflammatory environment MB inhibits cGMP production via blocking eNOS. The leukocyte transendothelial migration is modulated in both *in vitro* prophylactic and post-inflammatory treatment with MB. Furthermore, MB provokes apoptosis of endothelial cells in a dose/time-dependent manner.

## Supporting Information

Figure S1 **FACS gating strategy to identify PBMC subtypes.** (**A**) Untreated and unstained control plotted in FSC versus SSC. (**B**) Granulocyte gating CD177-FITC versus CD14-APC. (**C**) Lymphocyte gating CD3-Brilliant Violet versus CD19-Pe-Cy7. (**D**) Monocyte gating CD14-APC versus CD16-PE.
(TIF)

Table S1 (DOCX)
