# Supplementary figures and images for: Methylene Blue Modulates Transendothelial Migration of Peripheral Blood Cells
Source: PLoS One. 2013 Dec 10;8(12):e82214. doi: 10.1371/journal.pone.0082214 (PMC3858277; doi:10.1371/journal.pone.0082214)

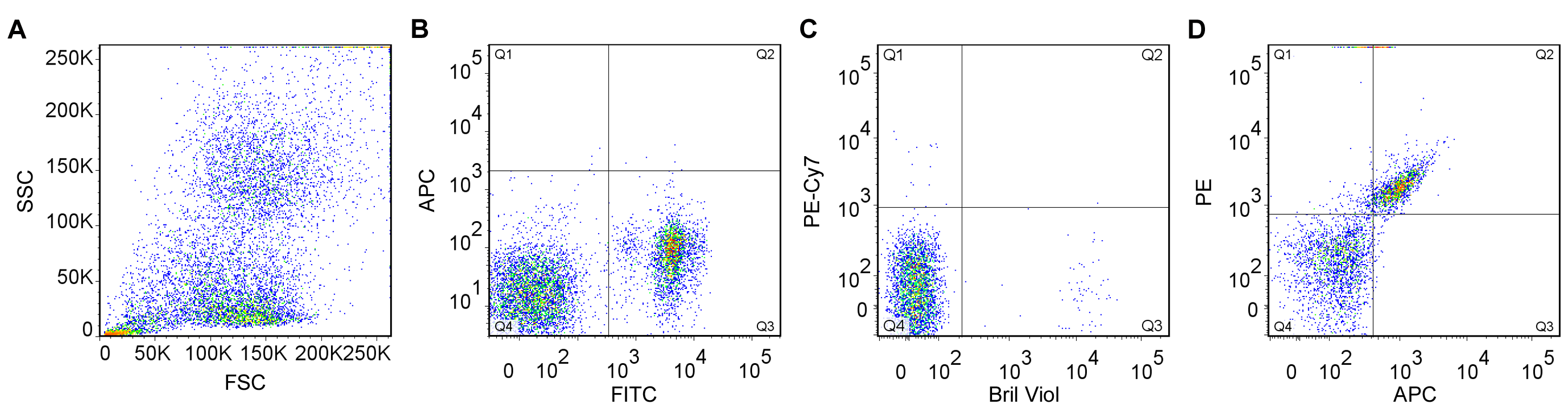

Supplement: Figure S1 — FACS gating strategy to identify PBMC subtypes. (A) Untreated and unstained control plotted in FSC versus SSC. (B) Granulocyte gating CD177-FITC versus CD14-APC. (C) Lymphocyte gating CD3-Brilliant Violet versus CD19-Pe-Cy7. (D) Monocyte gating CD14-APC versus CD16-PE. (TIF) [file pone.0082214.s001.tif]
