# Supplementary material for: Methylene Blue Modulates Transendothelial Migration of Peripheral Blood Cells
Source: PLoS One. 2013 Dec 10;8(12):e82214. doi: 10.1371/journal.pone.0082214 (PMC3858277; doi:10.1371/journal.pone.0082214)
Supplement: Table S1 — (DOCX) [file pone.0082214.s002.docx]

Table S1. Schematic summary of human microvascular endothelial cells treatment procedure.

| **Group** | **Concentration** | **Time [min]** | **Concentration** | **Time [min]** |
| --- | --- | --- | --- | --- |
|  | **MB** | |  |  |
| **MB** | 10µM | 30 |  |  |
|  |  | 120 |  |  |
|  | 30µM | 30 |  |  |
|  |  | 120 |  |  |
|  | 60µM | 30 |  |  |
|  |  | 120 |  |  |
|  | **LPS** | |  |  |
| **LPS** | 100µg/ml | 60 |  |  |
|  | **MB** | | **LPS** | |
| **MBLPS** | 10µM | 30 | 100µg/ml | 60 |
|  |  | 120 |  |  |
|  | 30µM | 30 | 100µg/ml | 60 |
|  |  | 120 |  |  |
|  | 60µM | 30 | 100µg/ml | 60 |
|  |  | 120 |  |  |
|  | **LPS** | | **MB** | |
| **LPSMB** | 100µg/ml | 60 | 10µM | 30 |
|  |  |  |  | 120 |
|  | 100µg/ml | 60 | 30µM | 30 |
|  |  |  |  | 120 |
|  | 100µg/ml | 60 | 60µM | 30 |
|  |  |  |  | 120 |

**Table S1. Schematic summary of human microvascular endothelial cells treatment procedure.** HuMEC-1 were treated either with methylene blue (MB), lipopolysaccharide (LPS), methylene blue followed by lipopolysaccharide (MBLPS) or lipopolysaccharide followed by methylene blue (LPSMB)
